# Supplementary material for: Genistein effect on cognition in prodromal Alzheimer’s disease patients. The GENIAL clinical trial
Source: Alzheimers Res Ther. 2022 Nov 4;14:164. doi: 10.1186/s13195-022-01097-2 (PMC9635167; doi:10.1186/s13195-022-01097-2)
Supplement: Supplementary file 1 — Additional file 1: Table S1. All possible comparisons of the values for the anterior cingulate gyrus before and after treatment with placebo or genistein. [file 13195_2022_1097_MOESM1_ESM.docx]

| Contrast | Estimate | Standard Error | Degrees of freedom | t ratio | p-value |
| --- | --- | --- | --- | --- | --- |
| After Genistein - Before Genistein | 0.05182 | 0.0697 | 17.0 | 0.744 | 0.8781 |
| After Genistein - After Placebo | 0.05625 | 0.1929 | 19.8 | 0.292 | 0.9911 |
| After Genistein - Before Placebo | 0.30250 | 0.1929 | 19.8 | 1568 | 0.4185 |
| Before Genistein - After Placebo | 0.00443 | 0.1929 | 19.8 | 0.023 | 1.0000 |
| Before Genistein - Before Placebo | 0.25068 | 0.1929 | 19.8 | 1299 | 0.5740 |
| After Placebo - Before Placebo | 0.24625 | 0.0817 | 17.0 | 3014 | 0.0357* |

**Table S1.- All possible comparisons of the values for the anterior cingulate gyrus before and after treatment with placebo or genistein.**
